# Ancient DNA sequence revealed by error-correcting codes

**DOI:** 10.1038/srep12051

**Published:** 2015-07-10

**Authors:** Marcelo M. Brandão, Larissa Spoladore, Luzinete C. B. Faria, Andréa S. L. Rocha, Marcio C. Silva-Filho, Reginaldo Palazzo

**Affiliations:** 1 Centro de Biologia Molecular e Engenharia Genética, Universidade Estadual de Campinas, Campinas, SP, Brazil; 2 Departamento de Genética, Escola Superior de Agricultura Luiz de Queiroz, Universidade de São Paulo, 13400-918, Piracicaba, SP, Brazil; 3 Departamento de Telemática, Faculdade de Engenharia Elétrica e de Computação, Universidade Estadual de Campinas, 13081-970, Campinas, SP, Brazil

## Abstract

A previously described DNA sequence generator algorithm (DNA-SGA) using error-correcting codes has been employed as a computational tool to address the evolutionary pathway of the genetic code. The code-generated sequence alignment demonstrated that a residue mutation revealed by the code can be found in the same position in sequences of distantly related taxa. Furthermore, the code-generated sequences do not promote amino acid changes in the deviant genomes through codon reassignment. A Bayesian evolutionary analysis of both code-generated and homologous sequences of the *Arabidopsis thaliana* malate dehydrogenase gene indicates an approximately 1 MYA divergence time from the MDH code-generated sequence node to its paralogous sequences. The DNA-SGA helps to determine the plesiomorphic state of DNA sequences because a single nucleotide alteration often occurs in distantly related taxa and can be found in the alternative codon patterns of noncanonical genetic codes. As a consequence, the algorithm may reveal an earlier stage of the evolution of the standard code.

Biological and digital communication systems have similarities with respect to the corresponding procedures used to convey the biological and digital information from one point to another, as well as in the data storage of digital media in a redundant array of independent disks (RAID)^1^ and the storage of genetic information in chromosomes. These similarities enable the use of algorithms in the modeling and analyses of biological systems and data. For instance, in eukaryotic cells, the information contained in the DNA is transmitted through RNA to produce the proteins needed at a precise moment and in specific compartments in the cell. Many enzymes and complex molecules coordinate their transport and are often assisted by protein intermediates in the cytosol and organellar membranes, thus identifying the correct location of a protein. In the same way, the transmission of flawless data through noisy channels in digital communication systems can be reliably achieved if, in addition to using an error-correcting code (ECC), extensive signal processing techniques are also employed^2^.

For quite some time there have been attempts to confirm the existence of an error-control mechanism in biological sequences similar to the ECC employed in digital sequences^3^, and although relevant, such studies have yet to provide a definitive answer. Recently our group developed an algorithm, known as DNA Sequence Generator Algorithm, which verifies whether a given DNA sequence can be identified as a codeword of an ECC. This goal was achieved when many distinct DNA sequences were identified as code words of G-linear codes (consisting of specific mappings and the underlying BCH codes)^4^,^5^,^6^,^7^ an important subclass of cyclic codes.

BCH codes were first proposed by Hocquenghem^8^ and independently rediscovered by Bose and Chaudhuri^9^; therefore, the acronym is made up of the initials of Bose, Chaudhuri, and Hocquenghem. When an underlying BCH code over Galois ring extension and/or Galois field extension identifies a given DNA sequence, two things may occur: 1) the given DNA sequence is a codeword of a G-linear code; or 2) it is a sequence belonging to the set of neighboring sequences differing by at least one nucleotide from the corresponding codeword of a G-linear code. This set of neighboring sequences is referred to as the “cloud” of a codeword.

When the DNA sequence generation algorithm identifies a DNA sequence belonging to the cloud of a codeword, it differs in a single nucleotide from the original sequence. Similar to biological DNA, this generated codeword may represent a silent mutation causing no effect on the translated amino acid or it may cause a residue change affecting for instance the protein structure and activity and consequently impairing its interactions with other proteins. Furthermore, the single nucleotide alteration can be restored, or equivalently, the codeword can be reverse engineered, returning it to its original sequence by applying one of the following algorithms: the Berlekamp-Massey decoding algorithm for codes over Galois field extensions^10^,^11^ or the Modified Berlekamp-Massey decoding algorithm for codes over Galois ring extensions^12^,^13^, together with the corresponding labeling associated with each analyzed sequence.

Recently, Ivanova and colleagues^14^ used a metagenomics approach to survey the prevalence of stop codon reassignment in naturally occurring microbial populations and proposed that the canonical genetic code may contain some deviations. Similarly, studies of the evolution of the genetic code have developed a hypothesis that differs from a frozen universal code^15^,^16^,^17^,^18^,^19^ and even the universality of the code^20^,^21^. It has been observed that each deviant genetic code contains codons that are associated with different amino acids and also with the canonical genetic code. Consequently, one may infer that such a process may have evolved from a standard code^16^. Such deviant genetic codes can be found in nuclear and mitochondrial genomes, in which mechanisms of codon reassignment have led to the differential reading of certain codons^22^,^23^,^24^. The evolution of the genetic code plays an important role in understanding the differences between the response of the DNA sequence identification process and the given DNA sequence because these differences can be related to either the canonical genetic code or to the several deviant genetic code^4^,^5^,^6^,^7^,^22^. In another example, Inomata and colleagues^25^ using multiple sequence alignment and test of neutrality, have demonstrated that a single replacement of guanine with adenine (position 926 of the gene) in *Drosophila melanogaster*, resulting on threonine at the 218 - amino acid position, was the ancestral form of the Gr5a gene in *D. melanogaster*^25^ and this single amino acid polymorphism (ALA218THR) represents a key impact on the trehalose sensitiveness.

The proposal of mathematical models describing such biological systems provides the needed tools for the development of systematic approaches for studies of mutations and polymorphisms and has applications in genetic engineering.

Thus, if an ECC can identify differences in a DNA sequence with a one-nucleotide resolution, the questions that should be addressed are as follows: if there is an error-correcting code underlying the DNA sequences, what are the biological implications regarding the single nucleotide (SNP) difference? And, is there a biological reasoning for such a difference? In the present study, we used the ECC approach proposed in references^4^,^5^,^6^,^7^ to evaluate whether the nucleotide difference between the original DNA sequence and the sequence identified as the codeword of the ECC is biologically significant in terms of evolution of this identified polymorphism.

## Results and Discussion

In this study the DNA sequence generation algorithm was applied as a computational tool to provide strong evidence of the evolution of the genetic code, in special on nucleotide and amino acid site specific polymorphism, by showing the existence of a mathematical structure underlying the actual DNA sequences and by investigating the real biological meaning of the difference in the specific position pointed out by the code-generated sequences.

The code-generated sequences that had a single nucleotide alteration, causing a residue change in the translated protein, were used in a Blastx analysis to verify if the alteration suggested by the ECC could be found in other sequences.

Analyses were run for the code generated sequences of the *Saccharomyces* YMR193 gene (GI 45269853), the *Triticum aestivum* wPR4 gene (GI 78096542), the *Nicotiana tabacum* antifungal CBP 20 gene (GI 632733), the *Citrus sinensis* chlorophyllase gene (GI 7328566), the *Arabidopsis thaliana* hevein-like protein PR4 gene (GI 186509758), the *Saccharomyces cerevisiae* OXA gene (GI 832917) and the *Homo sapiens* F1F0 ATP-synthase gene (GI 12587). These sequences are shown in Table S01.

As a result of this search approach, a number of different genes were found to contain the same nucleotide at the same altered position suggested by the error-correcting code, see Tables 1 and S02.

In some of the results, the suggested polymorphism could be found in DNA sequences of taxa that were closely related to the query sequence. For example, for the code-generated sequence of the YMR 193 gene from *Saccharomyces cerevisiae* (Tables S02a and S02b), a mitochondrial protein involved in the large ribosomal subunit, the same residue was also found in other Ascomycota sequences. The results for the code-generated antifungal CPB 20 gene from *Nicotiana tabacum* were similar to those from other eudicots. The results for the chlorophyllase gene in *Citrus sinensis* were also found in sequences in *Populus* spp. And the code-generated sequence for the OXA gene, which is involved in cytochrome oxidase biogenesis in *Saccharomyces cerevisiae,* showed the same residue in the altered position in other ascomycete sequences. However, different cases were found as well, such as the F1F0 ATP-synthase gene from *Homo sapiens*, in which the code-generated polymorphism of His to Gln could only be found at the same position in certain fungi sequences, a very distantly related taxa to *H. sapiens*, which may instead provide evidence that this algorithm may be describing ancient site specific sequences in which evolution acted to influence the current appearance of the gene. This was also observed in the wPR4 gene, which is involved in vacuolar defense in *Triticum aestivum*, in which the residue alterations in the positions suggested by the algorithm were found in eudicots, monocots, and other more distant taxa. The code-generated nucleotide sequence of the hevein-like protein PR4 of *Arabidopsis thaliana* showed an alteration that was also found in eudicots and monocots. Based on these results, one may infer the possibility that the ECC generated sequence might represent a plesiomorphic state of the SNP on DNA sequences of interest, and, may be viewed as an alternative generator of the canonical genetic code.

**Figure 1:**
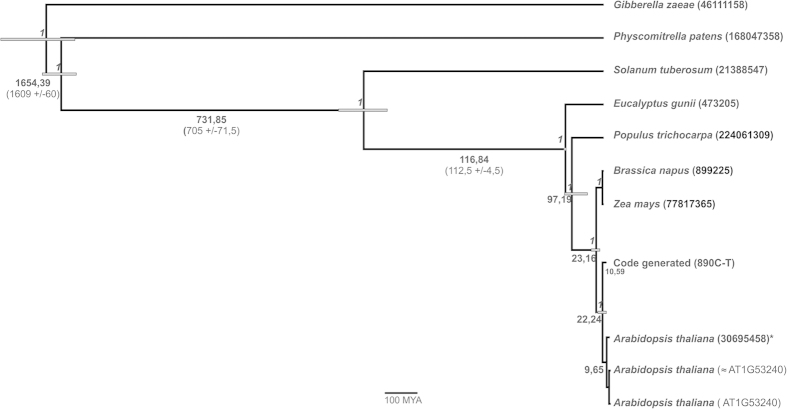
The malate dehydrogenase (MDH) phylogenetic proposal and date inference used to estimate the divergence time among malate dehydrogenase (MDH) sequences. The number on the nodes indicates the posterior probability, and the number along the length of the branch indicates the age in millions of years ago (MYA). The asterisk indicates the sequence analyzed by the G-linear code.

This SNP plesiomorphic state evidence is supported by a Bayesian analysis and divergence time calculation for the code-generated and homologous sequences of the *Arabidopsis thaliana* malate dehydrogenase gene. The analyses showed that the *A. thaliana* malate dehydrogenase sequences form a monophyletic group rooted in the sequence generated by the ECC (Fig. 1). This sequence was generated by the Klein-linear code ((1023, 1013, 3) BCH code over Z_4_ with the generator polynomial g(x) = x^10^ + x^9^ + x^8^ + 3x^7^ + x^6^ + x^4^ + x^3^ + 3x+1 and labeling C, Tables 2 and 3) and was recovered as an external group for *A. thaliana* clade (Fig. 1). The divergence time analysis indicates that the MDH code-generated sequence node has diverged approximately 1 MYA before the development of any *A. thaliana* paralogous sequences. These observations suggest that the sequence generated by the code might be more closely related to the ancestor of *A. thaliana* malate dehydrogenase rather than to other paralogous genes, evidencing that the ECC code generated sequence has a SNP that may be indicating the ancient state of this sequence. The application of an ECC does not aim to reconstruct full ancestral sequences from a given phylogenetic tree and aligned gene sequences of some current species; here we describe an ancestral site specific reconstruction based solely on DNA primary structure recovered from coding and decoding gene sequences.

Among the analyzed sequences, we identified several single nucleotide polymorphisms that were pointed out by the ECC as leading to a codon alteration (and also an amino acid alteration in the translated sequence), but in the deviant genetic codes, these altered codons correspond to the same amino acids that were found in the original sequence^15^,^26^,^27^.

In noncanonical genetic codes, alterations in the components of the translation mechanism confer different meanings to specific codons. For example, TGA is read as Trp^17^,^27^,^28^,^29^,^30^,^31^,^32^,^33^,^34^,^35^,^36^,^37^,^38^, AGA as Ser^33^,^34^,^35^,^36^,^37^, ATA as Met^17^,^31^,^35^,^37^,^38^,^39^,^40^, and TGA as Cys^17^,^23^.

When the F1 ATPase gene from *Ipomoea batatas* (GI 217937) was applied to the DNA Sequence Generator Algorithm, the output sequence presented an alteration in the sense codon TGG (encoding Trp) to become the stop codon TGA (Table 4a). A similar example is observed in the BRCA1 gene sequence in *H. sapiens* (GI 25140446), which is altered by the code from Cys to a stop codon (Table 4b). Interestingly, in the mitochondrial genetic code of most organisms, aside from green plants, the codon TGA is associated with tryptophan, and studies have shown that in the primary structure of mitochondrial and nuclear genomes, the TGA codon does not signal for the release of the transcription factors but instead codes for Trp^17^,^28^,^30^,^38^.

The code generated sequence for the Allergen Pol d5 gene of *Polistes dominulus* (GI 51093376) showed an alteration from AGT (Ser) to AGA (Arg), the same happening with the code generated sequence for the anti-epilepsy peptide precursor of *Mesobuthus martensii* (GI 16740522) showed an alteration from ATG (Met) to ATA (Ile) (Table 4b,c). In noncanonical genetic codes, AGA codes for Ser^33^,^34^,^35^,^36^,^37^ and ATA for Met^17^,^35^,^37^,^39^,^40^,^41^; therefore, these alterations could modify the folding and activity of the subsequent protein due to a change in the charge and hydropathy of the residues.

Often times, the same codon reassignment may independently occur multiple times in different taxa. The mechanisms leading to codon reassignment have yet to be fully elucidated and may be due to factors such as codon disappearance, an ambiguous intermediate, or unassigned codons^16^,^17^,^42^,^43^,^44^. Deviant genetic codes are an example of how populations cross over maladaptive valleys from one adaptive peak to another, in respect to error minimization, via adaptive bridges^45^. Therefore, the algorithm may underlie any of the stages of information transmission, representing an earlier stage of the evolution of the universal/canonical code.

The characters, character states, and the evolution of ancient genes or proteins can hardly be directly studied, because such molecule are rarely preserved over the evolutionary time or from any ancestral, living or preserved, has not been gathered from the nature. Pauling and Zuckerkandl once proposed that ancestral molecules could one day be “resurrected” by digging out from the evolution their ancient form^46^. Since then, different methods of ancestral sequence reconstruction (ASR) have emerged based on parsimony^47^, Bayesian inference^48^ or maximum likelihood^49^. Independently of the methodology used all these approaches rely on multiple sequence alignment with the aim of elucidating the complete and distant sequences (Supplementary material 1 presents a maximum likelihood analysis for the *Arabidopsis thaliana* Malate Dehydrogenase). Here, we hypothesize that the G-linear code may identify the original molecular primary structure of the sequence using only the intrinsic nucleotide composition. The DNA sequence generation algorithm can describe the plesiomorphic state of certain DNA character state sequences, as the suggested single nucleotide alteration often occurs in distant taxa and is maintained by alternative codon patterns in noncanonical genetic codes.

In summary, the G-linear code, commonly associated with reliable digital transmission, even with all the constraints inherent to the construction of the ECC^4^,^5^,^6^,^7^, unwraps the molecular component of every living cell when it is applied to the primary structure of DNA, thus revealing ancient information that may have been silenced by assorted evolutionary pressures that have shaped the present forms of life. This code generates point mutations that can be found in actual (real) sequences, and the DNA sequence generation algorithm can be used in computer simulations for the analysis of polymorphisms and mutations.

## Methods

### Identification of the DNA sequences

Although several DNA-encoding sequences (organelle-targeting sequences, introns, protein motifs, and full proteins) were identified by the corresponding G-linear codes over finite Galois rings and fields, as shown in Table 1, the majority of these DNA sequences were identified by the G-linear codes over rings. One possible explanation is that the latter algebraic structure may be more flexible than the algebraic structure of fields. As a consequence, the sequences identified by the corresponding G-linear codes over fields exhibit less adaptability than those offered by G-linear codes over rings. This observation suggests that it is possible to classify the proteins according to their stability in the mutation index, allowing a new approach for the classification of DNA sequences from a mathematical point of view.

All of the DNA sequences analyzed by the DNA sequence generation algorithm were identified as belonging to the “cloud” of the corresponding code words of the ECC. In other words, the actual DNA sequences differ from the corresponding code words of the ECC by a single nucleotide. The code-generated sequences in which the single nucleotide alteration led to an amino acid change in the translated protein were further analyzed.

These code-generated sequences were used as queries in a Blastx search, with the results filtered for green plants, fungi, bacteria, Archaea, algae and monocots from the NCBI non-redundant protein sequence database. The Blastx results were then aligned with Muscle^50^,^51^ (CLC Bio Genomics workbench plugin) and the position of the altered amino acid was compared with these results.

Several codons with the same meaning have been reassigned in independent lineages, which could mean that there is an underlying predisposition towards certain reassignments^43^. As an example of how the DNA sequence generation algorithm could be determining the ancient codon patterns in the analyzed species, we searched for codons in the code-generated sequences that were related to meaningful biological parts of nuclear or mitochondrial genomes^16^,^27^ and had a codon reassignment in other species.

### Evolutionary proposal and estimation of the divergence time based on Bayes approach - Estimates of divergence time among malate dehydrogenase (MDH) sequences

The divergence time between fungi and green plants^52^, mosses and vascular plants^52^,^53^ and eudicot rosids and asterids^54^ was used to estimate a divergence time for the *Arabidopsis thaliana* group. Species-level phylogenies were generated using a Bayesian uncorrelated lognormal relaxed clock model in Beast version 1.4.8^55^. The dataset followed the GTR + Γ model of substitution implemented in Beast, and two Monte Carlo Markov chains were run for 90,000,000 generations, using the Yule speciation model, using a 10% burn-in with sampling trees generated every 10,000 generations.

## Additional Information

**How to cite this article**: Brandão, M. M. *et al.* Ancient DNA sequence revealed by error-correcting codes. *Sci. Rep.* **5**, 12051; doi: 10.1038/srep12051 (2015).

## Supplementary Material

Supplementary Information

## Figures and Tables

**Table 1:** Polynomial-based DNA sequences generated by BCH codes over Galois ring and field extensions.

**Tables**	**DNA sequences**	**GI number**	**Organism**	**L R-F**	**Primitive polynomial**	**Generator polynomial**
S02.a)	TS	45269853	Sc	D/F	x^3^+ax^2^+bx+b	x^6^+x^5^+1
S02.b)	TS	45269853	Sc	B/R	x^6^+x^4^+x^3^+x+1	x^6^+2x^5^+x^4^+x^3^+3x+1
S02.c)	TS*	78096542	Ts	D/F	x^3^+bx^2^+x+a	x^6^+x^5^+x^4^+x+1
S02.d)	TS*	78096542	Ts	C/R	x^6^+x^5^+x^4^+x+1	x^6^+x^5^+x^4^+2x^2^+3x+1
S02.e)	TS*	632733	Nt	A/R	x^6^+x^5^+x^2^+x+1	x^6^+3x^5^+2x^4^+x^2^+x+1
S02.f)	TS*	632733	Nt	A/R	x^6^+x^5^+x^3^+x^2^+1	x^6^+3x^5^+x^3^+x^2^+2x+1
S02.g)	TS	7328566	Cs	B/R	x^6^+x^5^+1	x^6^+3x^5^+2x^3^+1
S02.h)	TS*	186509758	At	A/R	x^6^+x^5^+x^3^+x^2^+1	x^6^+3x^5^+x^3^+x^2^+2x+1
S02.i)	PM	832917	Sc	A/R	x^6^+x^5^+x^2^+x+1	x^6^+3x^5^+2x^4^+x^2^+x+1
S02.j)	TS	12587	Hs	C/R	x^6^+x^5^+1	x^6^+3x^5^+2x^3^+1
2a and b	**?**	30695458	At	C/R	x^10^+x^9^+x^8^+x^7^+x^6^+x^4^+x^3^+x+1	x^10^+x^9^+x^8^+3x^7^+x^6^+x^4^+x^3^+3x+1
3.a)	TS	217937	Ib	B/R	x^3^+ax^2^+ax+a	x^6^+x^5^+x^3^+x^2^+1
3.b)	TS	51093376	Pd	D/F	x^3^+ax^2^+bx+b	x^6^+x^5^+1
3.c)	TS	16740522	Mm	A/R	x^6^+x^5^+x^4^+x+1	x^6^+x^5^+x^4^+2x^2^+3x+1
3.d)	**?**	25140446	Hs	B/R	x^6^+x^5^+1	x^6^+3x^5^+2x^3^+1

Abbreviations: TS targeting sequence; PM protein motifs; L labelings A, B, C and D; R ring; F field; *signal or transit peptide without experimental evidence. Sc *Saccharomyces cerevisiae*; Ts *Triticum aestivum*; Nt *Nicotiana tabacum;* Cs *Citrus sinensis*; At *Arabidopsis thaliana*; Hs *Homo sapiens*; Ib *Ipomoea batatas*; Pd *Polistes dominulus*; Mm *Mesobuthus martensii.*

**Table 2:**
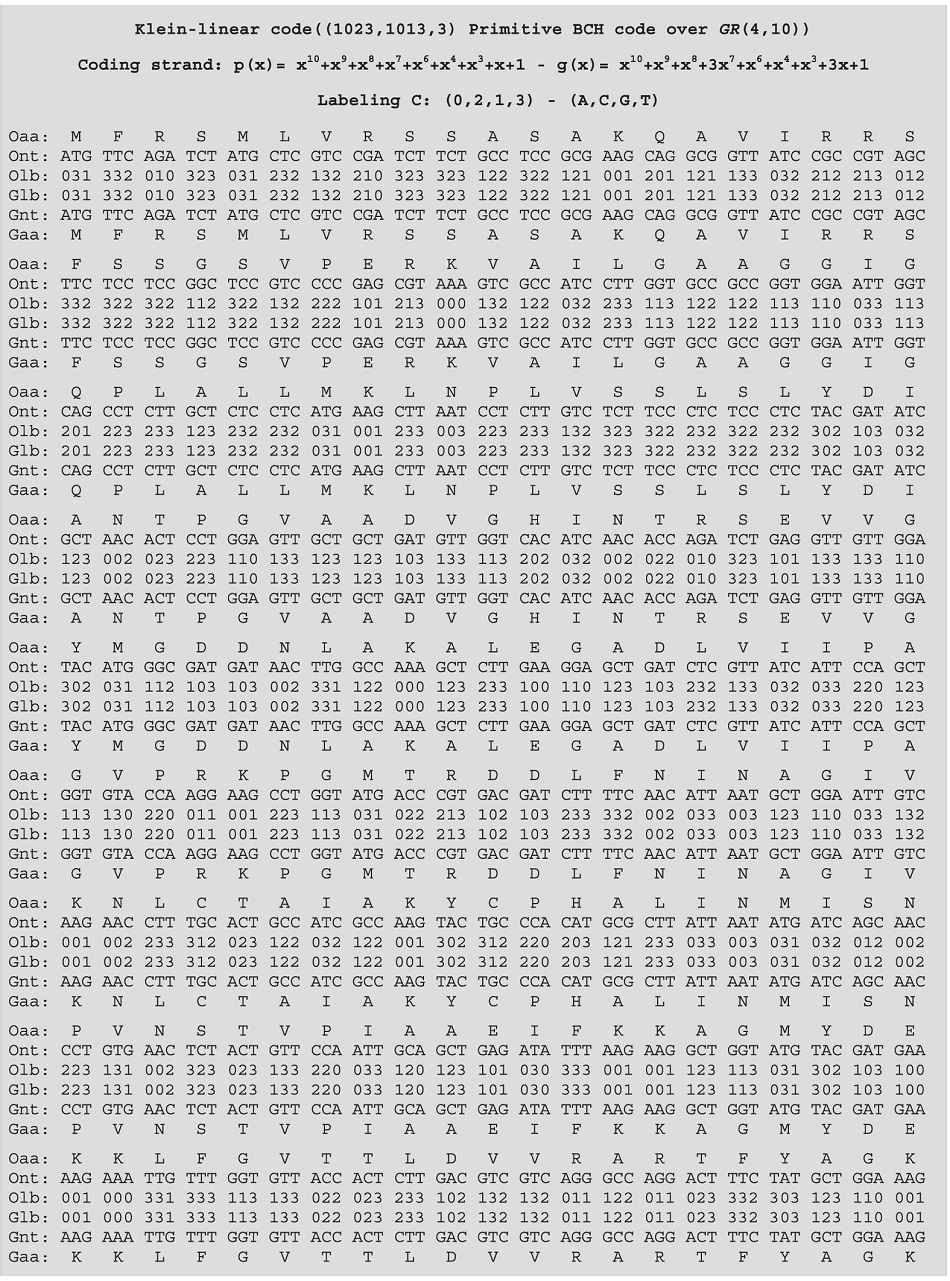
*A. thaliana* - Mitochondrial - Malate dehydrogenase 1 – GI number 30695458.

**Table 3:**
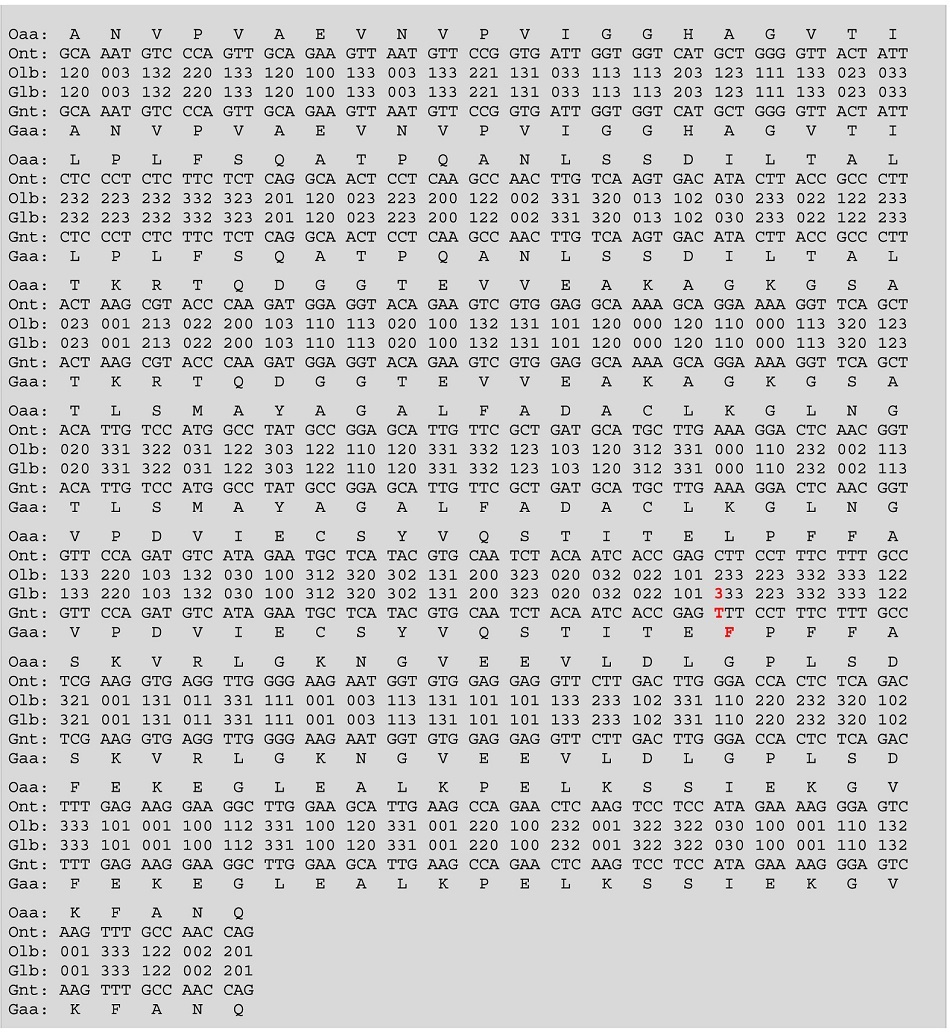
*A. thaliana* - Mitochondrial - Malate dehydrogenase 1 – GI number 30695458.

**Table 4:**
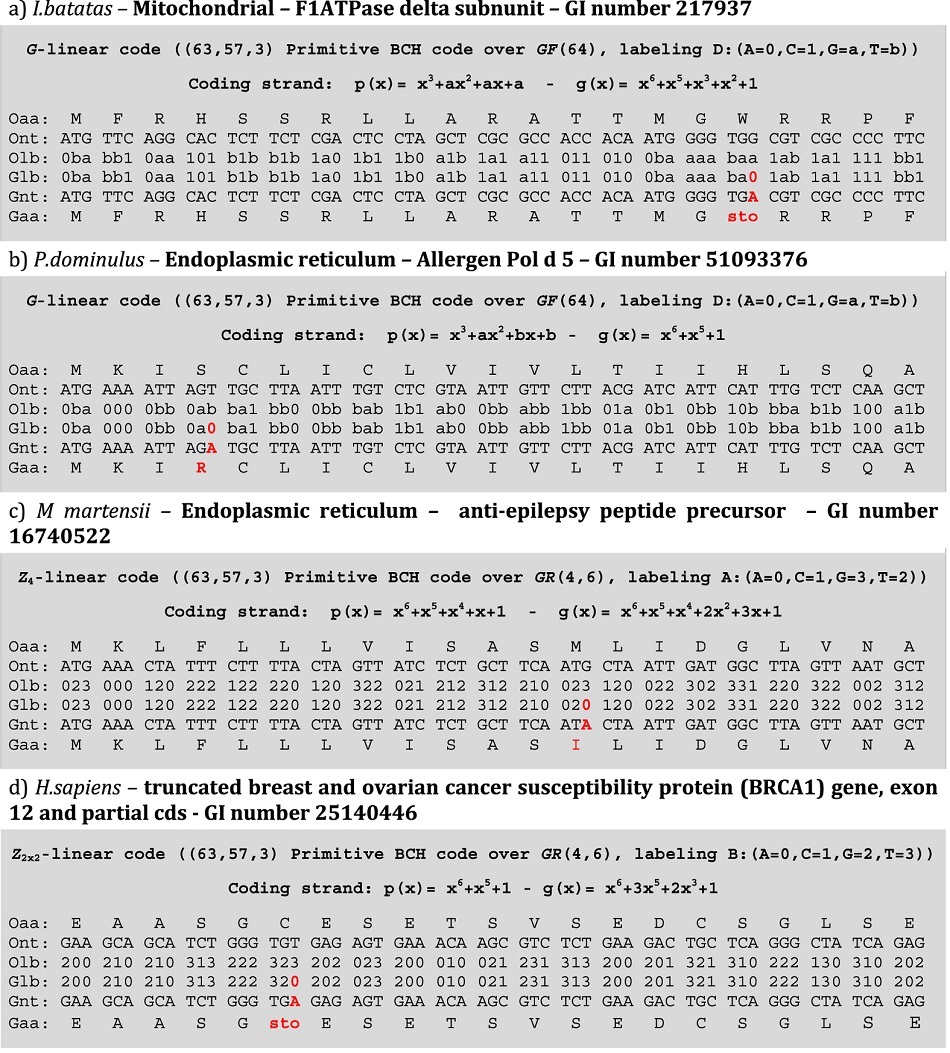
DNA sequences generated by BCH code.

## References

[b1] Patterson D. A., Gibson G. & Katz R. H. A case for redundant arrays of inexpensive disks (RAID). SIGMOD Rec 17, 109–116 (1988).

[b2] Benedetto S., Biglieri E. & Castellani V. Digital transmission theory. (Prentice-Hall, 1987).

[b3] MacWilliams F. J. The theory of error correcting codes / F.J. MacWilliams, N.J.A. Sloane. (North-Holland Pub. Co. ; sole distributors for the U.S.A. and Canada, Elsevier/North-Holland, 1977).

[b4] Faria L. C. B. *et al.* Is a genome a codeword of an error-correcting code? PLoS One 7, e36644 (2012). 22649495 10.1371/journal.pone.0036644 PMC3359345

[b5] Faria L. C. B., Rocha A. S. L. & Palazzo R. Jr. Transmission of intra-cellular genetic information: A system proposal. Journal of theoretical biology 358, 208–231 (2014). 24928152 10.1016/j.jtbi.2014.05.040

[b6] Faria L. C. B., Rocha A. S. L., Kleinschmidt J. H., Palazzo R. & Silva-Filho M. C. DNA sequences generated by BCH codes over GF(4). Electronics Letters 46, 203–204 (2010).

[b7] Rocha A. S. L., Faria L. C. B., Kleinschmidt J. H., Palazzo R. Jr. & Silva-Filho M. C. DNA sequences generated by Z_4_-linear codes. 2010 IEEE International Symposium on Information Theory Proceedings (ISIT). 1320–1324 (2010).

[b8] Hocquenghem A. Codes correcteurs d’erreurs. Chiffres 2, 147–156 (1959).

[b9] Bose R. C. & Ray-Chaudhuri D. K. On a class of error correcting binary group codes. Information and Control 3, 68–79 (1960).

[b10] Berlekamp E. R. Algebraic coding theory. (McGraw-Hill, 1968).

[b11] Massey J. L. Shift-Register Synthesis and Bch Decoding. Ieee T Inform Theory 15, 122–127 (1969).

[b12] Elia M., Interlando J. C. & Palazzo R. Computing the reciprocal of units in Galois rings. Journal of Discrete Mathematical Sciences and Cryptography 3, 41–55 (2000).

[b13] Interlando J. C, Palazzo R. J. & Elia M. On the decoding of Reed-Solomon and BCH codes over integer residue rings. Ieee T Inform Theory 43, 1013–1021 (1997).

[b14] Ivanova N. N. *et al.* Stop codon reassignments in the wild. Science 344, 909–913 (2014). 24855270 10.1126/science.1250691

[b15] Crick F. H. The origin of the genetic code. J Mol Biol 38, 367–379 (1968). 4887876 10.1016/0022-2836(68)90392-6

[b16] Knight R. D., Freeland S. J. & Landweber L. F. Rewiring the keyboard: evolvability of the genetic code. Nat Rev Genet 2, 49–58 (2001). 11253070 10.1038/35047500

[b17] Osawa S. & Jukes T. H. Codon reassignment (codon capture) in evolution. J Mol Evol 28, 271–278 (1989). 2499683 10.1007/BF02103422

[b18] Ōsawa S. Z. Evolution of the genetic code. (Oxford University Press, 1995).

[b19] Yokobori S., Suzuki T. & Watanabe K. Genetic code variations in mitochondria: tRNA as a major determinant of genetic code plasticity. J Mol Evol 53, 314–326 (2001). 11675591 10.1007/s002390010221

[b20] Jukes T. H. & Osawa S. Evolutionary changes in the genetic code. Comp Biochem Physiol B 106, 489–494 (1993). 8281749 10.1016/0305-0491(93)90122-l

[b21] Anderson S. *et al.* Sequence and organization of the human mitochondrial genome. Nature 290, 457–465 (1981). 7219534 10.1038/290457a0

[b22] Kawahara-Kobayashi A. *et al.* Simplification of the genetic code: restricted diversity of genetically encoded amino acids. Nucleic Acids Res 40, 10576–10584 (2012). 22909996 10.1093/nar/gks786 PMC3488234

[b23] Lozupone C. A., Knight R. D. & Landweber L. F. The molecular basis of nuclear genetic code change in ciliates. Curr Biol 11, 65–74 (2001). 11231122 10.1016/s0960-9822(01)00028-8

[b24] Yokogawa T. *et al.* Serine tRNA complementary to the nonuniversal serine codon CUG in Candida cylindracea: evolutionary implications. Proc Natl Acad Sci U S A 89, 7408–7411 (1992). 1502151 10.1073/pnas.89.16.7408 PMC49719

[b25] Inomata N. A Single-Amino-Acid Change of the Gustatory Receptor Gene, Gr5a, Has a Major Effect on Trehalose Sensitivity in a Natural Population of Drosophila melanogaster. Genetics 167, 1749–1758 (2004). 15342513 10.1534/genetics.104.027045 PMC1471011

[b26] Sengupta S., Yang X. & Higgs P. G. The mechanisms of codon reassignments in mitochondrial genetic codes. J Mol Evol 64, 662–688 (2007). 17541678 10.1007/s00239-006-0284-7 PMC1894752

[b27] Swire J., Judson O. P. & Burt A. Mitochondrial genetic codes evolve to match amino acid requirements of proteins. J Mol Evol 60, 128–139 (2005). 15696375 10.1007/s00239-004-0077-9

[b28] HayashiIshimaru Y., Ehara M., Inagaki Y. & Ohama T. A deviant mitochondrial genetic code in prymnesiophytes (yellow-algae): UGA codon for tryptophan. Curr Genet 32, 296–299 (1997). 9342410 10.1007/s002940050280

[b29] Turmel M. *et al.* The complete mitochondrial DNA sequences of Nephroselmis olivacea and Pedinomonas minor: Two radically different evolutionary patterns within green algae. Plant Cell 11, 1717–1729 (1999). 10488238 10.1105/tpc.11.9.1717 PMC144307

[b30] Boyen C., Leblanc C., Bonnard G., Grienenberger J. M. & Kloareg B. Nucleotide-Sequence of the Cox3 Gene from Chondrus-Crispus - Evidence That Uga Encodes Tryptophan and Evolutionary Implications. Nucleic Acids Res 22, 1400–1403 (1994). 8190631 10.1093/nar/22.8.1400 PMC307997

[b31] Macino G., Coruzzi G., Nobrega F. G., Li M. & Tzagoloff A. Use of the Uga Terminator as a Tryptophan Codon in Yeast Mitochondria. P Natl Acad Sci USA 76, 3784–3785 (1979). 10.1073/pnas.76.8.3784 PMC383918 226981

[b32] Beagley C. T., Okimoto R. & Wolstenholme D. R. The mitochondrial genome of the sea anemone Metridium senile (Cnidaria): Introns, a paucity of tRNA genes, and a near-standard genetic code. Genetics 148, 1091–1108 (1998). 9539427 10.1093/genetics/148.3.1091 PMC1460033

[b33] Bessho Y., Ohama T. & Osawa S. Planarian Mitochondria .2. The Unique Genetic-Code as Deduced from Cytochrome-C-Oxidase Subunit-I Gene-Sequences. J Mol Evol 34, 331–335 (1992). 1314909 10.1007/BF00160240

[b34] Telford M. J., Herniou E. A. & Russell R. B., Littlewood DTJ. Changes in mitochondrial genetic codes as phylogenetic characters: Two examples from the flatworms. P Natl Acad Sci USA 97, 11359–11364 (2000). 10.1073/pnas.97.21.11359 PMC17205 11027335

[b35] Hoffmann R. J., Boore J. L. & Brown W. M. A novel mitochondrial genome organization for the blue mussel, Mytilus edulis. Genetics 131, 397–412 (1992). 1386586 10.1093/genetics/131.2.397 PMC1205014

[b36] Jacobs H. T., Elliott D. J., Math V. B. & Farquharson A. Nucleotide sequence and gene organization of sea urchin mitochondrial DNA. J Mol Biol 202, 185–217 (1988). 3172215 10.1016/0022-2836(88)90452-4

[b37] Boore J. L., Daehler L. L. & Brown W. M. Complete sequence, gene arrangement, and genetic code of mitochondrial DNA of the cephalochordate Branchiostoma floridae (Amphioxus). Mol Biol Evol 16, 410–418 (1999). 10331267 10.1093/oxfordjournals.molbev.a026122

[b38] Barrell B. G., Bankier A. T. & Drouin J. A different genetic code in human mitochondria. Nature 282, 189–194 (1979). 226894 10.1038/282189a0

[b39] Clarkwalker G. D. & Weiller G. F. The Structure of the Small Mitochondrial-DNA of Kluyveromyces Thermotolerans Is Likely to Reflect the Ancestral Gene Order in Fungi. J Mol Evol 38, 593–601 (1994). 8083884 10.1007/BF00175879

[b40] Ehara M., HayashiIshimaru Y., Inagaki Y. & Ohama T. Use of a deviant mitochondrial genetic code in yellow-green algae as a landmark for segregating members within the phylum. J Mol Evol 45, 119–124 (1997). 9236270 10.1007/pl00006210

[b41] Kruft V., Eubel H., Jansch L., Werhahn W. & Braun H. P. Proteomic approach to identify novel mitochondrial proteins in Arabidopsis. Plant Physiol 127, 1694–1710 (2001). 11743114 PMC133574

[b42] Schultz D. W., Yarus M. & Transfer R. N. A. mutation and the malleability of the genetic code. J Mol Biol 235, 1377–1380 (1994). 8107079 10.1006/jmbi.1994.1094

[b43] Schultz D. W. & Yarus M. On malleability in the genetic code. J Mol Evol 42, 597–601 (1996). 8662012 10.1007/BF02352290

[b44] Sengupta S. & Higgs P. G. A unified model of codon reassignment in alternative genetic codes. Genetics 170, 831–840 (2005). 15781705 10.1534/genetics.104.037887 PMC1450412

[b45] Seaborg D. M. Was Wright right? The canonical genetic code is an empirical example of an adaptive peak in nature; deviant genetic codes evolved using adaptive bridges. J Mol Evol 71, 87–99 (2010). 20711776 10.1007/s00239-010-9373-8 PMC2924497

[b46] Pauling L., Zuckerkandl E., Henriksen T. & Lövstad R. Chemical Paleogenetics. Molecular “Restoration Studies” of Extinct Forms of Life. Acta Chemica Scandinavica 17 supl, 9–16 (1963).

[b47] Maddison W. P. Calculating the Probability Distributions of Ancestral States Reconstructed by Parsimony on Phylogenetic Trees. Systematic Biology 44, 474–481 (1995).

[b48] Schultz Gact R. The Role of Subjectivity in Reconstructing Ancestral Character States: A Bayesian Approach to Unknown Rates, States, and Transformation Asymmetries. Systematic Biology 48, 651–664 (1999).

[b49] Yang Z. & Roberts D. On the use of nucleic acid sequences to infer early branchings in the tree of life. Mol Biol Evol 12, 451–458 (1995). 7739387 10.1093/oxfordjournals.molbev.a040220

[b50] Edgar R. C. MUSCLE: multiple sequence alignment with high accuracy and high throughput. Nucleic Acids Res 32, 1792–1797 (2004). 15034147 10.1093/nar/gkh340 PMC390337

[b51] Edgar R. C. MUSCLE: a multiple sequence alignment method with reduced time and space complexity. BMC Bioinformatics 5, 113 (2004). 15318951 10.1186/1471-2105-5-113 PMC517706

[b52] Hedges S. B., Blair J. E., Venturi M. L. & Shoe J. L. A molecular timescale of eukaryote evolution and the rise of complex multicellular life. BMC evolutionary biology 4, 2 (2004). 15005799 10.1186/1471-2148-4-2 PMC341452

[b53] Heckman D. S., Geiser D. M., Eidell B. R., Stauffer R. L., Kardos N. L. & Hedges S. B. Molecular evidence for the early colonization of land by fungi and plants. Science 293, 1129–1133 (2001). 11498589 10.1126/science.1061457

[b54] Sanderson M. J., Thorne J. L., Wikstrom N. & Bremer K. Molecular evidence on plant divergence times. Am J Bot 91, 1656–1665 (2004). 21652315 10.3732/ajb.91.10.1656

[b55] Drummond A. J. & Rambaut A. BEAST: Bayesian evolutionary analysis by sampling trees. BMC evolutionary biology 7, 214 (2007). 17996036 10.1186/1471-2148-7-214 PMC2247476

