# Supplementary material for: Ancient DNA sequence revealed by error-correcting codes
Source: Sci Rep. 2015 Jul 10;5:12051. doi: 10.1038/srep12051 (PMC4498232; doi:10.1038/srep12051)
Supplement: Supplementary Information [file srep12051-s1.pdf]

SUPPLEMENTARY MATERIAL

Ancient DNA sequence revealed by error-correcting codes

Marcelo M. Brandão[1,2], Larissa Spoladore[1], Luzinete C. B. Faria[3], Andréa S. L. Rocha[3], Marcio C. Silva-Filho*[1], Reginaldo Palazzo Jr.*[3]

[1]Departamento de Genética, Escola Superior de Agricultura Luiz de Queiroz, Universidade de São Paulo, 13400-918, Piracicaba, SP, Brazil

[2]Centro de Biologia Molecular e Engenharia Genética, Universidade Estadual de Campinas, Campinas, SP, Brazil

[3]Departamento de Telemática, Faculdade de Engenharia Elétrica e de Computação, Universidade Estadual de Campinas, 13081-970, Campinas, SP, Brazil

* Corresponding author contact information:

Marcio C. Silva-Filho: mdcsilva@usp.br

Reginaldo Palazzo Jr: palazzo@dt.fee.unicamp.br

Table S01: Sequences used on this work showing the alteration identified by the code and it's respectively blast analyses. The alteration is represented by the amino acid changes due to the nucleotide alteration.

Species	Alteration (TableS02)	Blast results
Saccharomyces YMR193 gene-mitochondrial protein of the large subunit (GI 45269853)	I→T (S02.a)	Neurospora crassa (85110139)
		Ashbya gossypii (45198359)
		Naumovozyma dairenensis (365990107)
	L→I (S02.b)	Eremothecium cymbalariae (363748975)
		Saccharomyces cerevisiae x Saccharomyces kudriavzevii (365758940)
		Scheffersomyces stipites (126133881)
Triticum aestivum wPR4 gene-vacuolar defense (GI 78096542)	A→S (S02.c)	Vitis vinifera (147844408)
		Vitis vinifera (225453020)
		Vitis vinifera (3511147)
		Vitis vinifera (225453020)
		Vitis pseudoreticulata (362097039)
		Ricinus communis (255580937)
		Sorghum bicolor (242068991)
	V→ L (S02.a)	Glycine max (356573113)
		Glycine max (255629027)
		Glycine max (356573103)
		Populus tremula x Populus alba (118138836)
		Populus trichocarpa (224123428)
Nicotiana tabacum – antifungal CBP 20 (GI 632733)	S→T (S02.d)	Medicago truncatula (388515239)
		Medicago truncatula (388509152)
		Capsicum annuum (6601327)
		Capsicum chinense (42557355)
	L→V (S02.e)	*Medicago truncatula* (388515239)
		Medicago truncatula (388509152)
		Glycine max (351725403)
Citrus sinensis – chlorophylase (GI 7328566)	V→A (S02.f)	*Populus trichocarpa* (224082682)
Arabidopsis thaliana – havein like protein	I→L (S02.g)	*Populus tremula* x *Populus alba* (118138836)

PR4 (GI 186509758)		*Populus trichocarpa* (224123428)
		Glycine max (255629027)
		Glycine max (356573103)
		Glycine max (356573113)
		Populus trichocarpa (224131254)
		Lotus japonicus (388520989)
		Solanum tuberosum (139699)
		Solanum lycopersicum (1888561)
		Capsicum chinense (42557355)
		Nicotiana tabacum (632736)
		Solanum tuberosum (139698)
		Capsicum annuum (6601327)
		Dioscorea bulbifera (2738609)
		Triticum aestivum (45862004)
		Triticum aestivum (78096543)
		Hordeum vulgare (326517637)
		Glycine max (351721773)
Saccharomyces cerevisiae OXA gene – cytochrome oxidase biogenesis (GI 832917)	G→ A (S02.h)	*Mycosphaerella graminicola* (398393476)
		Aspergillus clavatus (121718317)
		Aspergillus fumigatus (70984948)
		Aspergillus fumigatus (159126095)
		Schizosaccharmyces japonicus (213408000)
		Yarrowia lipolytica (50557420)
		Ogataea parapolymorpha (320581780)
		Millerozyma farinosa (359384038)
		Millerozyma farinosa (359384620)
		Debaryomyces hansenii (50405829)
		Kluyveromyces lactis (50307981)
		Tuber melanosporum (296425195)
		Spathaspora passalidarum (344299573)
Homo sapiens F1F0 ATP-synthase (GI 12587)	H→Q (S02.i)	*Fomitiporia mediterranea* (393217450)
		Punctularia strigosozonata (390602282)
		Neosartorya fischeri (119497145)
		Aspergillus fumigatus (70990422)

		Aspergillus clavatus (121703117)
		Aspergillus flavus (238483405)
		Cordyceps militaris (346324930)
		Ajellomyces dermatitidis (261196376)
		Paracoccidioides brasiliensis (225677807)
		Paracoccidioides sp. '*lutzii*' (295663535)

Table S02: DNA sequences generated by BCH code

a) *S. cerevisiae* – **Mitochondrial – 54S ribosomal protein – GI number 45269853**

Coding strand: $p(x) = x^3+ax^2+bx+b$ – $g(x) = x^6+x^5+1$

labeling D: (0,1,a,b) – (A,C,G,T)

Oaa:	M	Q	K	I	F	R	P	F	Q	L	T	R	G	F	T	S	S	V	K	N	F
Ont:	ATG	CAA	AAA	ATT	TTC	AGA	CCA	TTC	CAA	TTA	ACG	AGA	GGC	TTT	ACC	TCT	TCC	GTA	AAA	AAC	TTC
Olb:	0ba	100	000	0bb	bb1	0a0	110	bb1	100	bb0	01a	0a0	aa1	bbb	011	b1b	b11	ab0	000	001	bb1
Glb:	0ba	100	000	01b	bb1	0a0	110	bb1	100	bb0	01a	0a0	aa1	bbb	011	b1b	b11	ab0	000	001	bb1
Gnt:	ATG	CAA	AAA	ACT	TTC	AGA	CCA	TTC	CAA	TTA	ACG	AGA	GGC	TTT	ACC	TCT	TCC	GTA	AAA	AAC	TTC
Gaa:	M	Q	K	**T**	F	R	P	F	Q	L	T	R	G	F	T	S	S	V	K	N	F

b) *S. cerevisae* – **Mitochondrial - 54S ribosomal protein – GI number 45269853**

Coding strand: $p(x) = x^6+x^4+x^3+x+1$ – $g(x) = x^6+2x^5+x^4+x^3+3x+1$

labeling B: (0,1,2,3) – (A,C,G,T)

Oaa:	M	Q	K	I	F	R	P	F	Q	L	T	R	G	F	T	S	S	V	K	N	F
Ont:	ATG	CAA	AAA	ATT	TTC	AGA	CCA	TTC	CAA	TTA	ACG	AGA	GGC	TTT	ACC	TCT	TCC	GTA	AAA	AAC	TTC
Olb:	032	100	000	033	331	020	110	331	100	330	012	020	221	333	011	313	311	230	000	001	331
Glb:	032	100	000	033	331	020	110	331	100	030	012	020	221	333	011	313	311	330	000	001	331
Gnt:	ATG	CAA	AAA	ATT	TTC	AGA	CCA	TTC	CAA	ATA	ACG	AGA	GGC	TTT	ACC	TCT	TCC	TTA	AAA	AAC	TTC
Gaa:	M	Q	K	I	F	R	P	F	Q	**I**	T	R	G	F	T	S	S	**L**	K	N	F

c) *T. aestivum* – **Endoplasmic reticulum – wPR4g gene for putative vacuolar defense protein – GI number 78096542**

Coding strand: $p(x) = x^3+bx^2+x+a$ – $g(x) = x^6+x^5+x^4+x+1$

labelling D: (0,1,a,b) – (A,C,G,T)

Oaa:	M	A	A	R	L	A	L	V	A	A	L	L	C	A	G	A	T	A	A	A	A
Ont:	ATG	GCC	GCA	CGC	CTC	GCG	CTG	GTG	GCG	GCG	CTC	CTG	TGC	GCC	GGT	GCC	ACG	GCC	GCC	GCG	GCG
Olb:	0ba	a11	a10	1a1	1b1	a1a	1ba	aba	a1a	a1a	1b1	1ba	ba1	a11	aab	a11	01a	a11	a11	a1a	a1a
Glb:	0ba	a11	a10	1a1	1b1	a1a	1ba	aba	a1a	a1a	1b1	1ba	ba1	b11	aab	a11	01a	a11	a11	a1a	a1a
Gnt:	ATG	GCC	GCA	CGC	CTC	GCG	CTG	GTG	GCG	GCG	CTC	CTG	TGC	TCC	GGT	GCC	ACG	GCC	GCC	GCG	GCG
Gaa:	M	A	A	R	L	A	L	V	A	A	L	L	C	**S**	G	A	T	A	A	A	A

d) *T. aestivum* – **Endoplasmic reticulum – wPR4g gene for putative vacuolar defense protein – GI number 78096542**

Coding strand: $p(x) = x^6+x^5+x^4+x+1$ – $g(x) = x^6+x^5+x^4+2x^2+3x+1$

labeling C: (0,2,1,3) – (A,C,G,T)

Oaa:	M	A	A	R	L	A	L	V	A	A	L	L	C	A	G	A	T	A	A	A	A
Ont:	ATG	GCC	GCA	CGC	CTC	GCG	CTG	GTG	GCG	GCG	CTC	CTG	TGC	GCC	GGT	GCC	ACG	GCC	GCC	GCG	GCG
Olb:	031	122	120	212	232	121	231	131	121	121	232	231	312	122	113	122	021	122	122	121	121
Glb:	031	122	120	212	232	121	231	331	121	121	232	231	312	122	113	122	021	122	122	121	121
Gnt:	ATG	GCC	GCA	CGC	CTC	GCG	CTG	TTG	GCG	GCG	CTC	CTG	TGC	GCC	GGT	GCC	ACG	GCC	GCC	GCG	GCG
Gaa:	M	A	A	R	L	A	L	**L**	A	A	L	L	C	A	G	A	T	A	A	A	A

e) *N. tabacum*– **Endoplasmic reticulum– Pathogen- and wound-inducible antifungal protein CBP20* – GI number 632733**

Coding strand: $p(x) = x^6+x^5+x^2+x+1$ – $g(x) = x^6+3x^5+2x^4+x^2+x+1$

labelling A: (0,1,3,2) – (A,C,G,T)

Oaa:	G	K	L	S	T	L	L	F	A	L	V	L	Y	V	I	A	A	G	A	N	A
Ont:	GGA	AAG	CTA	AGT	ACA	CTT	TTA	TTT	GCT	CTG	GTC	CTC	TAT	GTC	ATA	GCC	GCA	GGA	GCT	AAT	GCA
Olb:	330	003	120	032	010	122	220	222	312	123	321	121	202	321	020	311	310	330	312	002	310
Glb:	330	003	120	012	010	122	220	222	312	123	321	121	202	321	020	321	310	330	312	002	310
Gnt:	GGA	AAG	CTA	ACT	ACA	CTT	TTA	TTT	GCT	CTG	GTC	CTC	TAT	GTC	ATA	GTC	GCA	GGA	GCT	AAT	GCA
Gaa:	G	K	L	**T**	T	L	L	F	A	L	V	L	Y	V	I	**V**	A	G	A	N	A

Table S02: DNA sequences generated by BCH code

f) *N. tabacum*– Endoplasmic reticulum– Pathogen- and wound-inducible antifungal protein CBP20* – GI number 632733

Coding strand: $p(x) = x^6+x^5+x^3+x^2+1$ – $g(x) = x^6+3x^5+x^3+x^2+2x+1$

labeling C: (0,2,1,3) – (A,C,G,T)

Oaa:	G	K	L	S	T	L	L	F	A	L	V	L	Y	V	I	A	A	G	A	N	A
Ont:	GGA	AAG	CTA	AGT	ACA	CTT	TTA	TTT	GCT	CTG	GTC	CTC	TAT	GTC	ATA	GCC	GCA	GGA	GCT	AAT	GCA
Olb:	110	001	230	013	020	233	330	333	123	231	132	232	303	132	030	122	120	110	123	003	120
Glb:	110	001	230	013	020	233	330	333	123	231	132	132	303	132	030	122	120	110	123	003	122
Gnt:	GGA	AAG	CTA	AGT	ACA	CTT	TTA	TTT	GCT	CTG	GTC	GTC	TAT	GTC	ATA	GCC	GCA	GGA	GCT	AAT	GCC
Gaa:	G	K	L	S	T	L	L	F	A	L	V	V	Y	V	I	A	A	G	A	N	A

g) ***C. sinensis*** – Chloroplast – Chlorophylase-1 – GI number 7328566

Coding strand: $p(x) = x^6+x^5+1$ – $g(x) = x^6+3x^5+2x^3+1$

labeling B: (0,1,2,3) – (A,C,G,T)

Oaa:	M	A	A	M	V	D	A	K	P	A	A	S	V	Q	G	T	P	L	L	A	T
Ont:	ATG	GCA	GCA	ATG	GTG	GAC	GCC	AAG	CCT	GCA	GCT	TCA	GTG	CAA	GGC	ACT	CCC	CTT	TTG	GCT	ACG
Olb:	032	210	210	032	232	201	211	002	113	210	213	310	232	100	221	013	111	133	332	213	012
Glb:	032	210	210	032	232	201	211	002	113	210	212	310	212	100	221	013	111	133	332	213	012
Gnt:	ATG	GCA	GCA	ATG	GTG	GAC	GCC	AAG	CCT	GCA	GCG	TCA	GCG	CAA	GGC	ACT	CCC	CTT	TTG	GCT	ACG
Gaa:	M	A	A	M	V	D	A	K	P	A	A	S	A	Q	G	T	P	L	L	A	T

h) *A. thaliana* – Mitochondrial – Pathogenesis related protein 4* - GI number 186509758

Coding strand: $p(x)= x^6+x^5+x^3+x^2+1$ – $g(x)= x^6+3x^5+x^3+x^2+2x+1$

labeling A: (0,1,3,2) – (A,C,G,T)

Oaa:	M	K	I	R	L	S	I	T	I	I	L	L	S	Y	T	V	A	T	V	A	G
Ont:	ATG	AAG	ATC	AGA	CTT	AGC	ATA	ACC	ATC	ATA	CTT	TTA	TCA	TAC	ACA	GTG	GCT	ACG	GTG	GCC	GGA
Olb:	023	003	021	030	122	031	020	011	021	020	122	220	210	201	010	323	312	013	323	311	330
Glb:	023	003	021	030	122	031	120	011	021	020	122	220	210	201	010	323	312	013	323	311	330
Gnt:	ATG	AAG	ATC	AGA	CTT	AGC	CTA	ACC	ATC	ATA	CTT	TTA	TCA	TAC	ACA	GTG	GCT	ACG	GTG	GCC	GGA
Gaa:	M	K	I	R	L	S	L	T	I	I	L	L	S	Y	T	V	A	T	V	A	G

i) *S. cerevisiae* - OXA 1 – protein motifs – GI number 832917

Coding strand: $p(x)= x^6+x^5+x^2+x+1$ – $g(x)= x^6+3x^5+2x^4+x^2+x+1$

labeling A: (0,1,3,2) – (A,C,G,T)

Oaa:	A	V	H	V	Y	S	G	L	P	W	W	G	T	I	A	A	T	T	I	L	I
Ont:	GCC	GTT	CAT	GTT	TAC	TCT	GGG	TTG	CCT	TGG	TGG	GGA	ACT	ATC	GCG	GCC	ACC	ACC	ATC	CTC	ATT
Olb:	311	322	102	322	201	212	333	223	112	233	233	330	012	021	313	311	011	011	021	121	022
Glb:	311	322	102	322	201	212	333	223	112	233	233	310	012	021	313	311	011	011	021	121	022
Gnt:	GCC	GTT	CAT	GTT	TAC	TCT	GGG	TTG	CCT	TGG	TGG	GCA	ACT	ATC	GCG	GCC	ACC	ACC	ATC	CTC	ATT
Gaa:	A	V	H	V	Y	S	G	L	P	W	W	A	T	I	A	A	T	T	I	L	I

j) *H. sapiens* – Mitochondrial – ATPase delta-subunit – GI number 12587

Coding strand: $p(x)= x^6+x^5+1$ – $g(x)= x^6+3x^5+2x^3+1$

labeling C: (0,2,1,3) – (A,C,G,T)

Oaa:	L	P	A	A	L	L	R	R	P	G	L	G	R	L	V	R	H	A	R	A	Y
Ont:	CTG	CCC	GCC	GCG	CTG	CTC	CGC	CGC	CCG	GGA	CTT	GGC	CGC	CTC	GTC	CGC	CAC	GCC	CGT	GCC	TAT
Olb:	231	222	122	121	231	232	212	212	221	110	233	112	212	232	132	212	202	122	213	122	303
Glb:	231	222	122	121	231	232	212	212	221	110	233	112	212	232	132	212	201	122	213	122	303
Gnt:	CTG	CCC	GCC	GCG	CTG	CTC	CGC	CGC	CCG	GGA	CTT	GGC	CGC	CTC	GTC	CGC	CAG	GCC	CGT	GCC	TAT
Gaa:	L	P	A	A	L	L	R	R	P	G	L	G	R	L	V	R	Q	A	R	A	Y

Abbreviations: Oaa= original amino acid, Ont= original nucleotideos, Olb= original labelling; Glb: generated labelling; Gnt: generated nucleotideo; Gaa: generated amino acid. Red: shows where the error occurred in the targeting sequences.
p(x)= primitive polynomial ; p(x)'= reciprocal polynomial of p(x).
g(x)= generator polynomial ; g(x)'= reciprocal generator polynomial of g(x).

Suplementary figure 1 - Annotated tree with ancestral node sequences present at the PAML_rst_FILE. The red clade present the code generated MDH_ATH sequence.

Supplementary files 1

CODEML analyses for ancestral sequence proposal of the *Arabidopsis thaliana* Malate Dehydrogenase (MDH_ATH) code generated sequence.

MDH_Plant_Bact.mlc = Contains many information on evolutionary rates of all sequences

PAML_rst_FILE = Contains ancestral states for sites and for all nodes

PAML_TREE.pdf = Annotated tree with ancestral node sequences present at the PAML_rst_FILE. The red clade present the code generated MDH_ATH sequence.

All the above files are zip compacted under the S_Files1.zip file and can be downloaded here https://goo.gl/1hLRQN.

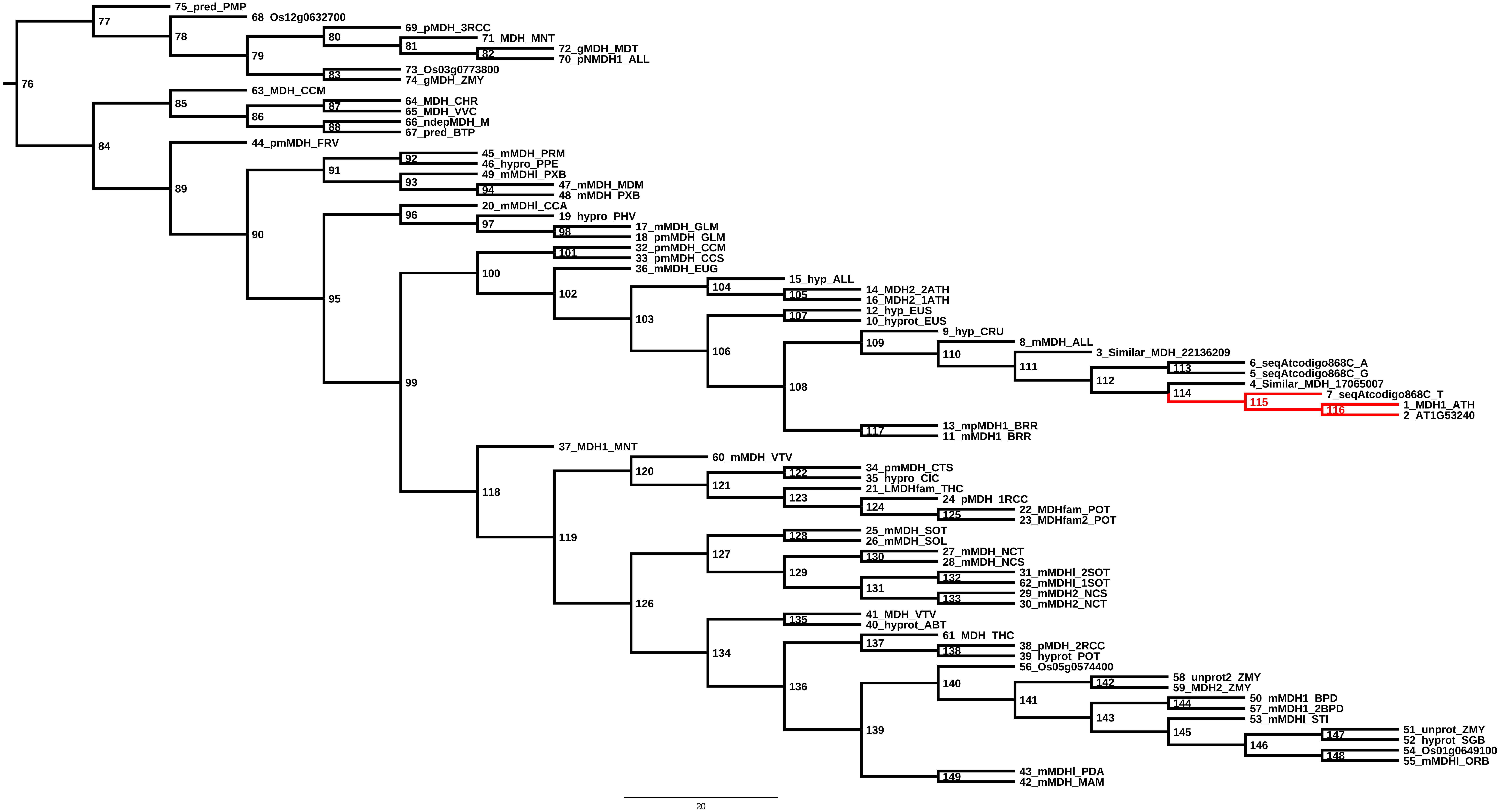
75_pred_PMP
68_Os12g0632700
69_pMDH_3RCC
71_MDH_MNT
72_gMDH_MDT
70_pNMDH1_ALL
73_Os03g0773800
74_gMDH_ZMY
63_MDH_CCM
64_MDH_CHR
65_MDH_VVC
66_ndepMDH_M
67_pred_BTP
44_pmMDH_FRV
45_mMDH_PRM
46_hypro_PPE
49_mMDHl_PXB
47_mMDH_MDM
48_mMDH_PXB
20_mMDHl_CCA
19_hypro_PHV
17_mMDH_GLM
18_pmMDH_GLM
32_pmMDH_CCM
33_pmMDH_CCS
36_mMDH_EUG
15_hyp_ALL
14_MDH2_2ATH
16_MDH2_1ATH
12_hyp_EUS
10_hyprot_EUS
9_hyp_CRU
8_mMDH_ALL
3_Similar_MDH_22136209
6_seqAtcodigo868C_A
5_seqAtcodigo868C_G
4_Similar_MDH_17065007
7_seqAtcodigo868C_T
1_MDH1_ATH
2_AT1G53240
13_mpMDH1_BRR
11_mMDH1_BRR
37_MDH1_MNT
60_mMDH_VTV
34_pmMDH_CTS
35_hypro_CIC
21_LMDHfam_THC
24_pMDH_1RCC
22_MDHfam_POT
23_MDHfam2_POT
25_mMDH_SOT
26_mMDH_SOL
27_mMDH_NCT
28_mMDH_NCS
31_mMDHl_2SOT
62_mMDHl_1SOT
29_mMDH2_NCS
30_mMDH2_NCT
41_MDH_VTV
40_hyprot_ABT
61_MDH_THC
38_pMDH_2RCC
39_hyprot_POT
56_Os05g0574400
58_unprot2_ZMY
59_MDH2_ZMY
50_mMDH1_BPD
57_mMDH1_2BPD
53_mMDHl_STI
51_unprot_ZMY
52_hyprot_SGB
54_Os01g0649100
55_mMDHl_ORB
43_mMDHl_PDA
42_mMDH_MAM
76
77
78
79
80
81
82
83
84
85
86
87
88
89
90
91
92
93
94
95
96
97
98
99
100
101
102
103
104
105
106
107
108
109
110
111
112
113
114
115
116
117
118
119
120
121
122
123
124
125
126
127
128
129
130
131
132
133
134
135
136
137
138
139
140
141
142
143
144
145
146
147
148
149
20
